# Agonizing GABA_B_R suppresses GLP-1RA’s chronotropic effect and reduces post-myocardial infarction arrhythmogenesis

**DOI:** 10.3389/fphar.2025.1616181

**Published:** 2025-10-31

**Authors:** Run Qi, Zhang Jingjing, Gu Hongchang, Li Chenyu, Hu He, Li Juan, Zhao Yuqin, Wu Xiaolin

**Affiliations:** ^1^ Department of Cardiology, Xiangyang Central Hospital, Affiliated Hospital of Hubei University of Arts and Science, Xiangyang, Hubei, China; ^2^ Institute of Cardiovascular Diseases, Xiangyang Central Hospital, Affiliated Hospital of Hubei University of Arts and Science, Xiangyang, Hubei, China; ^3^ Hubei Key Laboratory of Biological Targeted Therapy. Union Hospital, Tongji Medical Collage, Huazhong University of Science and Technology, Wuhan, Hubei, China; ^4^ Department of Cardiology, Renmin Hospital of Wuhan University, Wuhan, Hubei, China; ^5^ Department of Electrophysiology, Cardiovascular Research Institute of Wuhan University, Wuhan, Hubei, China; ^6^ Department of Electrophysiology, Hubei Key Laboratory of Cardiology, Wuhan, Hubei, China

**Keywords:** glucagon-like-peptide 1 receptor, GABAB receptor, heart rate, myocardial infarction, ventricular arrhythmias

## Abstract

**Background:**

Glucagon-like peptide-1 receptor agonists (GLP-1RAs) have been reported to improve cardiovascular outcomes, potentially through glucose metabolism-independent mechanisms. However, their mechanism of heart rhythm remains controversial.

**Methods:**

We investigated the role of the GABA_B_ receptor (GABA_B_R) in mediating GLP-1RA’s chronotropic and anti-arrhythmic effects in a murine myocardial infarction (MI) model. MI was induced by left anterior descending artery ligation. Cardiomyocyte-specific *Gabbr1*-knockout (*Gabbr1* ^cKO^) mice were generated via AAV9-cTnT-Cre delivery to *Gabbr1* ^f/f^ mice. Cardiac sympathetic denervation was achieved by 6-hydroxydopamine (6-OHDA) treatment and sympathectomy. Mechanistic insights were obtained through Western blotting, immunofluorescence, *in vivo* electrophysiology, and patch-clamp recordings.

**Results:**

GLP-1RA increased the heart rate independent of the sympathetic input, suggesting a cardiac-autonomous mechanism. GABA_B_R activation attenuated GLP-1RA-induced tachycardia, whereas Gabrb1 deficiency exacerbated it. GABA_B_R agonism enhanced resistance to ventricular arrhythmias post-MI in a GLP-1RA-dependent manner. Patch-clamp analysis revealed that GABA_B_R-induced repolarization can be suppressed by semaglutide in a dose-dependent manner, indicating the possible mechanism.

**Conclusion:**

GABA_B_R activation counteracts GLP-1RA’s chronotropic effects while synergistically enhancing anti-arrhythmic efficacy post-MI, highlighting a novel GABA_B_R/GLP-1R interaction in cardiac electrophysiology.

## 1 Introduction

Glucagon-like peptide-1 receptor agonists (GLP-1RAs), the first-line drugs for type II diabetes mellitus and obesity, also improve cardiac contractility and cardiometabolism, and prevent ischemia-induced myocardial injury via coupling with the cAMP/PKA and PI3K/Akt pathways in clinical practice (Ussher and Drucker, 2023; Dang et al., 2025). Paradoxically, GLP-1 RAs have been found to increase the heart rate (HR), which is thought to be a safety concern, as an elevated HR is an independent risk factor for cardiovascular adverse events (Hozawa et al., 2004). However, GLP-1RAs’ positive chronotropic effect—particularly sinus tachycardia—remains mechanistically unresolved. The earliest speculation was that the positive chronotropic effect was secondary to a reduction in blood pressure mediated by vessel smooth muscle relaxation and sodium excretion. However, this hypothesis was dismissed because the acute increase in the HR post-GLP-1RA treatment was not accompanied with blood pressure decrease. The GLP-1RA mechanism affecting the HR is explained by the following: (I) autonomous nervous system modulation due to the compromised parasympathetic nervous activity after infusion of a GLP-1RA, exendin-4 and (II) a direct GLP-1 receptor-mediated effect on the endogenous sinoatrial pacemaker node of the heart (Lorente et al., 2000). Mounting evidence supports that GLP-1RAs increase the HR in a cardiac-autonomous manner rather than through autonomous nerve or baroreflex modulation (Jakob and Krieglstein, 1997; Woo et al., 2013; Hill et al., 2024).

The GABA_B_R, which is a class C metabotropic G protein-coupled receptor, mediates slow and long-lasting neuronal synapse inhibition through indirect K^+^ and Ca^2+^ channel gating and through other second messengers such as cAMP. Central nervous (e.g., hypothalamus and nucleus tractus solitarius) GABAnergic neurons are suppressed by GLP-1R signaling, whereas GABAnergic activation has been well established to decrease the HR (Wang et al., 2001; Lu et al., 2024; Bony et al., 2013). Moreover, cloning has demonstrated high cardiac content of GABA_B_R, which triggers inward-rectifying K^+^ currents (GIRK), accelerating repolarization and stabilizing membrane potential (Fortin et al., 2020; Francois et al., 2025). Otherwise, depletion of GABA_B_R on cardiomyocytes prolonged the action potential duration (APD), creating electrophysiological heterogeneity that may predispose to arrhythmias (Gähwiler and Brown, 1985). Given that GLP-1R is expressed in cardiomyocytes, we hypothesize that the GLP-1R counteracts with GABA_B_R on cardiomyocytes. To this end, we co-activated GABA_B_R/GLP-1R and found that compared to single GLP-1RA, the post-MI ventricular arrhythmias was improved possibly via potentiation of GIRK. It is suggested that GLP-1RAs increase the HR through a cardiomyocyte-autonomous mechanism, independent of sympathetic input—challenging prior assumptions and offering novel insights into anti-arrhythmogenesis mechanism post-myocardial infarction (MI).

## 2 Methods

### 2.1 Animal and animal treatment

Our study examined male mice because male animals exhibited less variability in myocardial infarction phenotypes and showed more significant resilience to arrhythmia after treatment with GLP-1R/GABA_B_R agonists. All animal experiment procedures followed the principles of the Guide for the Care and Use of Laboratory Animals published by the US National Institutes of Health (NIH publication no. 85-23, revised 1996) and were authorized by the Animal Care and Use Committee of Renmin Hospital of Wuhan University under the approval number: 20220301A. All male Sprague–Dawley rats (8–9 weeks old) and male C57BL/6J mice (8–10 weeks old) were purchased from Shulaibao (Wuhan) Biotechnology Co., Ltd. *Gabbr1* flox mice (#S-CKO-11747) were on a C57B/J background. The myocardial infarction models were induced by 6-0 suture ligation of the left anterior descending branch of the coronary artery. Sham surgery was performed in the same procedure, except for artery ligation. The success in myocardial infarction modeling is indicated by ST segment elevation from ECG. Semaglutide was injected subcutaneously at a dose of 100 μg/kg/day and treated once a day. Stellate ganglionectomy was performed on all mice under a surgical microscope, following the procedure previously reported (Hill et al., 2024). Lungs were pulled caudally to visualize stellate ganglion between the first and second rib beneath the parietal pleura. Chemical denervation was achieved via 100 mg/kg 6-hydroxydopamine (6-OHDA, diluted into 0.3% ascorbic acid) injection. Baclofen (15 mg/kg) dissolved in 0.9% saline was applied interperitoneally 1 day before semaglutide treatment. Mice were fed for another 1 week after surgery. All mice were fed in a standard environment with controlled light/dark cycles (12-h light/12-h dark), ambient temperature, and humidity. Tail-vein injection of AAV9-cTnT-Cre virus (WZ Biosciences, Inc, Shandong, China, 0.5 × 10^E11^ GC/pup) was performed to achieve cardiomyocyte-specific knockout of *Gabbr1*. All mice were grouped and sacrificed randomly, but no blind test was conducted in the animal experimental procedures.

### 2.2 Cell culture and cell treatment

iPSC-derived cardiomyocyte was obtained from CardioEasy^®^ (CA2201106, CellAPY, China). The cells were cultured in DMEM/F-12 (11554546, Gibco, United States), 2% B27 insulin-free (A1895601, Thermo Fisher Scientific, United States) penicillin–streptomycin (1%; 100 units/mL penicillin and 100 μg/mL streptomycin) (Cellclone; Genetix Biotech Asia Pvt. Ltd.). The cell culture incubator (Forma™ Steri-Cycle™, 370, Thermo Fisher, USA) was in a humidified air containing 5% CO_2_ at 37 °C. A total of 1×10^6^ cells were seeded in a T-25 culture flask (Eppendorf, Hamburg, Germany). Each plate was seeded with an equal amount of cells.

### 2.3 Echocardiography

The cardiac function of mice was evaluated at 1 week after surgery with echocardiography (Visual et al., 2100, Toronto, Canada), equipped with a 23-MHz line array transducer. The mice were maintained under 0.5% anesthesia and placed in the supine position on a 37 °C heating pad. M-mode images were obtained to measure left ventricular end-systolic volume (LVESV) and left ventricular end-diastolic volume (LVEDV). Left ventricular ejection fraction (LVEF) and fractional shortening (FS) were obtained from the VEVO system.

### 2.4 Histopathology analysis

The hearts were fixed in 4% paraformaldehyde and sectioned into 5 μm-thick slices. Based on the standard procedure, the hematoxylin–eosin (HE) staining and Masson’s staining in cross-section were performed to evaluate myocardial condition and fibrosis condensation. ImageJ (Fiji) was used for the calculation.

### 2.5 Immunofluorescence

The ventricular sections were cut into approximately 5-µm slices, followed by paraffinization, rehydration, heat-mediated antigen retrieval, and treatment with 3% H_2_O_2_. After blocking for 1 h with 5% bovine serum, the slices were incubated with the primary antibody overnight at 4 C. Antibody information was as follows: anti-tyrosine hydroxylase (Abcam, ab6211, Germany, 1:500) and anti-cTnT (Abcam, ab8295, Germany, 1:1000). The next day, sections were incubated with the horseradish peroxidase (HRP)-labeled secondary antibody for 2 h at 37 C. Immunofluorescence images were captured using a confocal laser scanning microscope (ZEISS LSM 800).

### 2.6 Electrocardiogram (ECG) and *in vivo* electrophysiology

Atrial pacing was produced through transesophageal programmed electrical stimulation. Mouse anesthesia was maintained by 1% isoflurane. Standard surface ECG was recorded using the PowerLab System (AD instruments) with a subcutaneous ECG surface (lead II). The tracheal tube was inserted into the trachea through the glottis, and the chest fluctuation of the mice was observed to be consistent with the ventilator frequency, which proved that the tracheal intubation was successful. Then, a 2.2F six-polar catheter was inserted into the esophagus near the left atrium, and correct placement was confirmed through burst waves. To correct for the HR, Bazett’s formula-corrected QT interval (QTc) was used. The ECG of mice at the rest state was recorded consecutively for 5–10 min. Subsequently, sodium pentobarbital (50 mg/kg, intraperitoneal) was used to anesthetize animals. With a platinum MAP electrode and stimulation procedures, the monophasic action potentials (MAPs) of the left ventricle were recorded. The paired platinum-stimulating electrode was positioned on the basal surface of the right ventricle to deliver regular pacing. The heart was stimulated with a regular pacing cycle length (PCL). Action potential duration 90 (APD_90_) was defined as the average repolarization time of 90% of 6–8 consecutive MAPs when the PCL was 150 ms. S1–S1 pacing was used to measure APD and activation latency time (ALT). To induce ALT, PCL was decreased, starting at 150 ms and gradually reduced by 10 ms, and then by 5 ms from 100 to 50 ms, until APD alternans occurred. Ventricular arrhythmias (VAs) were induced by burst pacing with 2 ms pulses delivered at 50 Hz for 2 s, repeated 20 times and separated by 2-s intervals. VA is defined as ventricular tachycardia (VT) or ventricular fibrillation (VF) lasting 2 s or more.

### 2.7 Enzyme-linked immunosorbent assay (ELISA)

Cardiac samples were homogenized in a tissue lysis buffer (pH: 7.4 with 150 mmol/L NaCl, 1% Triton ×100, and proteinase inhibitor) and centrifuged (10,000 g, 10 min, 4 °C). The supernatant was then collected, and its protein concentration was adjusted to 500 μg/μL. Commercial ELISA kits were used to detect the norepinephrine level (ab287789, Abcam, USA).

### 2.8 Patch clamp recording

A soft glass capillary pulled to a tip resistance of 1.5–2 MΩ (Sutter Instruments, Novato, CA) was used for whole-cell patch clamp. Signals were recorded using an Axopatch 200A (Axon Instruments, Foster City, CA) with a computer-interacted 125-kHz Labmaster board (Axon Instruments). Membrane currents were sampled at 1–2 kHz and filtered at 2 kHz. Series resistance (*R*_s_ = 7.0 ± 1.0 MΩ) was compensated by ≈ 80%. Cells to be tested were incubated at 35 °C in the bath solution supplemented with 300 ng/mL pertussis toxin for 4 h. The buffer was supplemented with 2 mM Co^2+^ and 3 mM 4-aminopyridine to block transient rectifying current and calcium current. Voltage commands, data acquisition, and analysis were performed using pClamp 6.0.

### 2.9 Western blotting (WB) analysis

The tissue samples were lysed in 1× RIPA buffer (G2002, ServiceBio, Wuhan, China). Subsequently, protein samples were separated using SDS-PAGE and then transferred into the PVDF membrane (IPVH00010, Merk Millipore, Germany). After the PVDF membranes were blocked with 5% fat-free bovine milk for 2 h, specific primary antibodies were incubated overnight at 4 °C. The primary antibodies included. The next day, the membranes were incubated with HRP-conjugated goat anti-rabbit secondary antibodies (ServiceBio, Wuhan, China) for 1 h. Finally, protein bands were visualized through enhanced chemiluminescence (BL523B, Biosharp, China). Antibody information was as follows: anti-αSMA (1:1000, Cell Signaling Technology, United Kingdom, #19245) and anti-TGF-β (1:1000, Cell Signaling Technology, United Kingdom, #3711).

### 2.10 Statistical analysis

All statistical analyses were performed using GraphPad Prism 8.0 (Inc., La Jolla, CA, USA). Data are presented as mean ± SD. Statistical analyses used repeated two-way ANOVA and paired Student’s *t*-tests where appropriate (*p* <0.05). Values were considered statistically significant when P < 0.05.

## 3 Results

### 3.1 GLP-1RA-mediated cardiac positive chronotropic effect is possibly not neuronal-dependent

**FIGURE 1:**
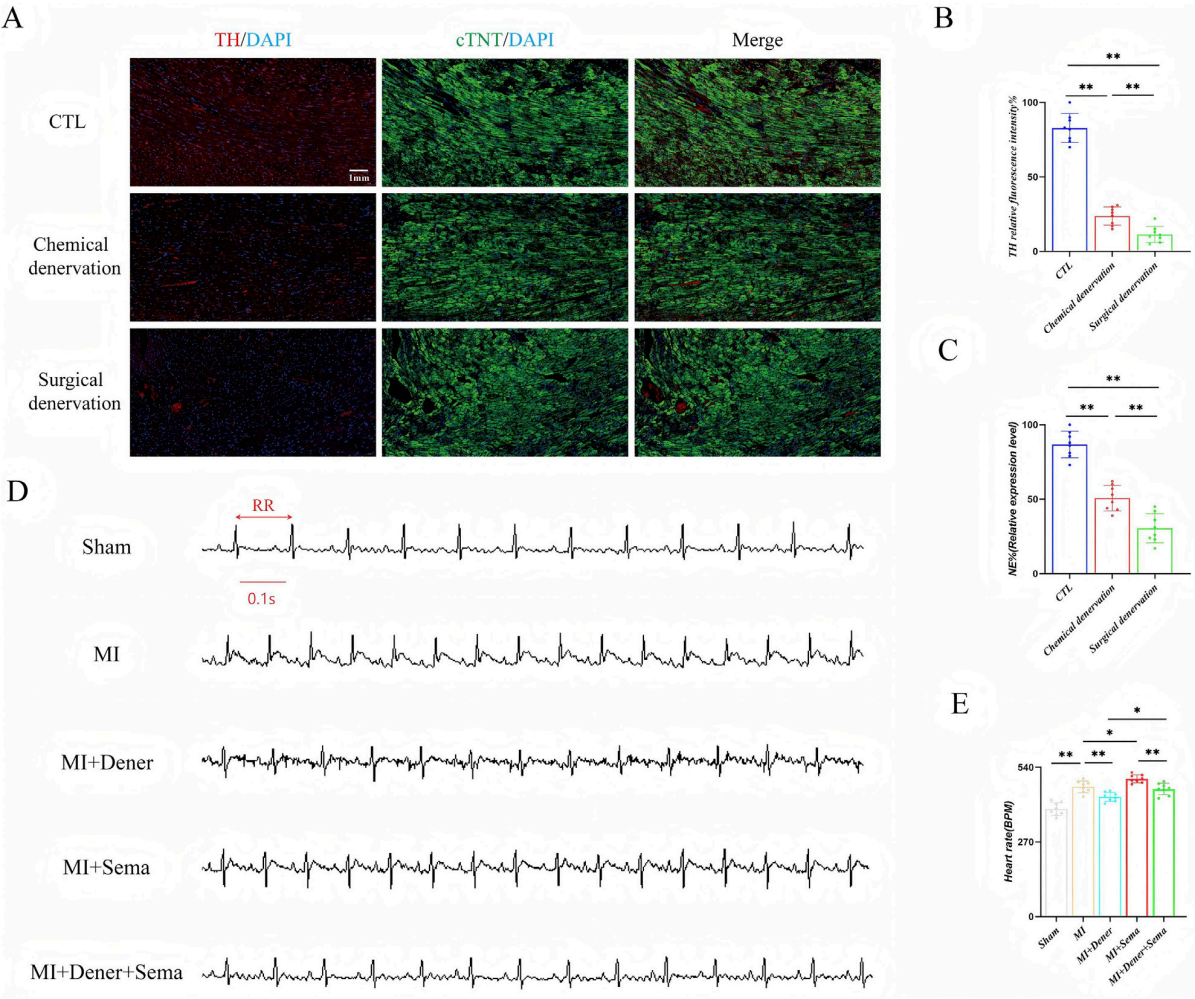
GLP-1RA-mediated cardiac positive chronotropic effect is possibly not neuronal-dependent. **(A)** The representative images of tyrosine hydroxylase immunofluorescent staining. **(B)** Quantitative analysis of relative tyrosine hydroxylase positive rate in all groups (n = 8 biological replicates). **(C)** Quantitative analysis of relative norepinephrine expression levels in all groups (n = 8 biological replicates). **(D)** Representative images of ECG. **(E)** Quantitative analysis of the HR in all groups (n = 8 biological replicates). Data are presented as mean ± SD. Differences among more than two groups were compared using ANOVA, followed by Tukey’s test. SD, standard deviation; CTL, control; MI, myocardial infarction; TH, tyrosine hydroxylase; cTNT: cardiac troponin; NE, norepinephrine; Dener, denervation; Sema, semaglutide; BPM, beat per minute. *P < 0.05 and **P < 0.01.

GLP-1R is highly enriched in autonomous neurons and cardiomyocytes. To investigate whether the autonomous nervous system is sufficient for mediating GLP-1RA-related fast pacing effect, we blocked cardiac sympathetic innervation via 6-OHDA infusion and surgical sympathectomy. 6-OHDA, a neurotoxic agent that degrades sympathetic nerve terminals, reduced nearly 75% of tyrosine hydroxylase (TH, a key enzyme involved in the production of norepinephrine) fluorescence signals in cardiac tissue, and bilateral stellate sympathectomy depleted 87% of TH^+^ signals (Figures 1A,B). The indicator of sympathetic innervation, norepinephrine, was also significantly reduced after both 6-OHDA infusion and sympathectomy, as determined by ELISA (Figure 1C). Semaglutide treatment increased the HR to a new baseline, whereas heart denervation did not lead to further change in the HR, which suggests that the autonomous nervous system might not be sufficient for GLP-1RA’s positive chronotropic effect (Figures 1D,E).

### 3.2 GABA_B_R activation improves the GLP-1RA-mediated heart tachycardia effect

**FIGURE 2:**
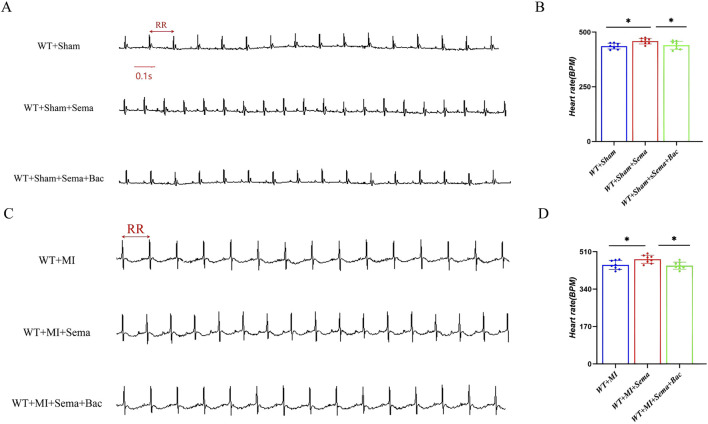
GABABR activation improves the GLP-1RA-mediated heart tachycardia effect. **(A,C)** Representative images of ECG. **(B,D)** Quantitative analysis of the HR in all groups (n = 8 biological replicates). Data are presented as mean ± SD. Differences among more than two groups were compared using ANOVA followed by Tukey’s test. SD, standard deviation; MI, myocardial infarction; BPM, beat per minute; Sema, semaglutide; Bac, baclofen. *P < 0.05 and **P < 0.01.

GABA_B_R was identified as a direct binding partner of GLP-1R in a previous biotin proximity labeling screen (Dang et al., 2025). To validate the function of the GABA_B_R/GLP-1R interaction, pharmacological activation of GABA_B_R with baclofen, a GABA-mimetic GABA_B_R agonist, prior to MI induction, reduced the chronotropic effect of semaglutide under physiological conditions (Figures 2A–D). However, the activation of GABA_B_R did not accelerate the HR under physiological conditions (Supplementary Figure S1). Moreover, this antagonistic effect of GLP-1RA and GABA_B_R on the HR was preserved in post-MI mice. Here, we hypothesize that GABA_B_R serves as an antagonizing mediator for GLP-1R regarding HR modulation, potentially through direct receptor cross-talk or intracellular downstream.

### 3.3 GLP-1R collaborates with GABA_B_R to increase the resilience to post-myocardial infarction ventricular arrhythmia

**FIGURE 3:**
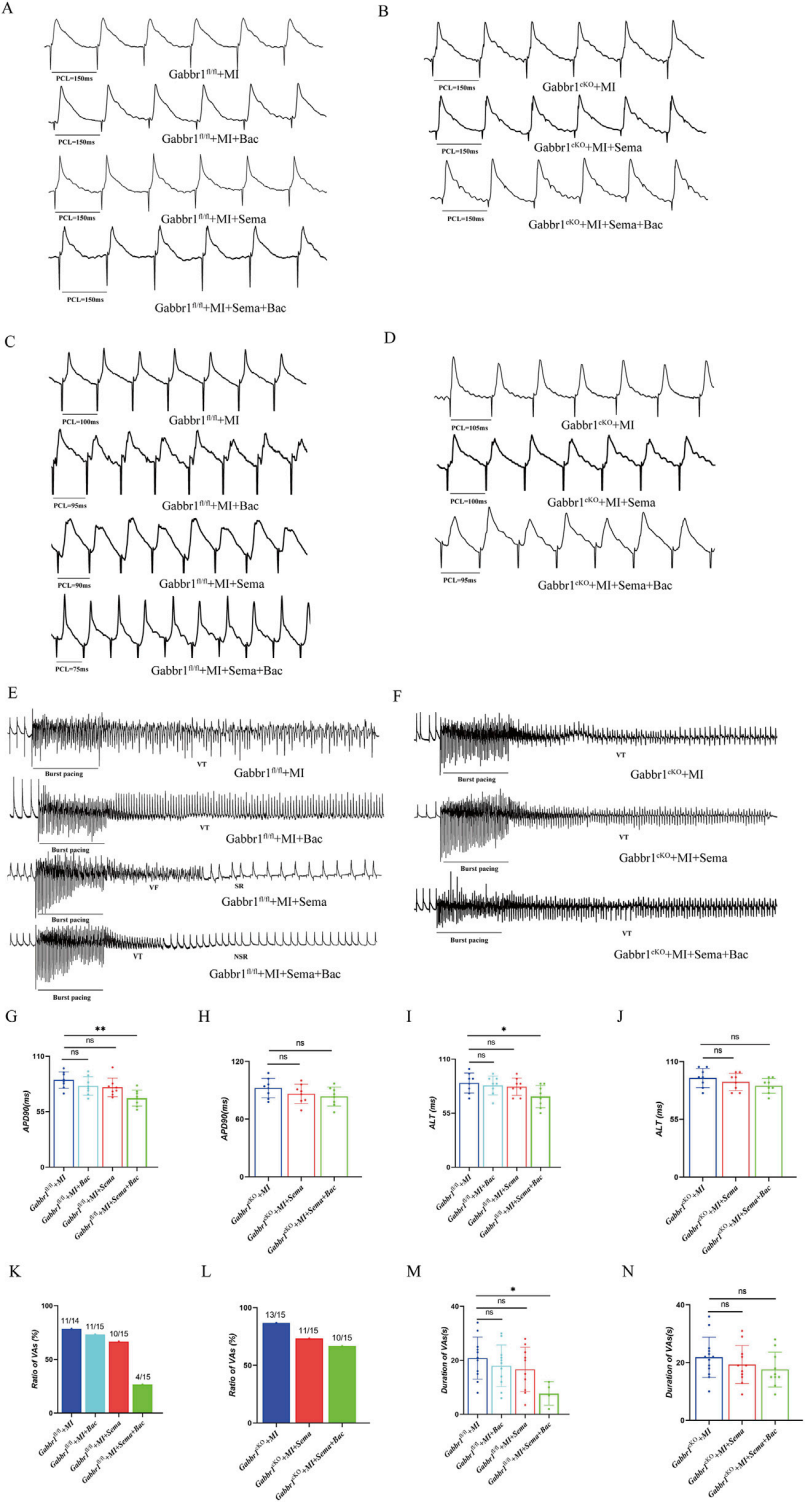
GLP-1R collaborates with GABABR to increase the resilience to post-myocardial infarction ventricular arrhythmia. **(A,B)** Representative images of the MAP recordings at a PCL of 150 ms; **(C,D)** representative images of the MAP recordings of ALT; **(E,F)** representative images of the MAP recordings after burst pacing; **(G,H)** quantitative analysis of APD90 at 150 ms PCL in all groups (n = 8 biological replicates); **(I,J)** quantitative analysis of the threshold interval for ALT in all groups (n = 8 biological replicates); **(K,L)** quantitative analysis of VA inducibility in all groups (n = 14–15 biological replicates); **(M,N)** quantitative analysis of duration of ventricular arrhythmias in all groups. MAP, monophasic action potentials; PCL, pacing cycle length; VAs, ventricular arrhythmias. Data are presented as mean ± SD. Differences among more than two groups were compared using ANOVA followed by Tukey’s test. SD, standard deviation; Sema, semaglutide; Bac, baclofen; VT, ventricular tachycardia; VF, ventricular fibrillation; SR, sinus rhythm; NSR, normal sinus rhythm. *P < 0.05 and **P < 0.01.

To determine whether the baclofen-induced reduction in the HR depends on GLP-1R signaling, we generated cardiac GABABR-knockout (*Gabbr1* ^cKO^) mice by injecting AAV9-cTnT-Cre into Gabbr1^f/f^ mice and assessed cardiac electrophysiology *in vivo* (Supplementary Figure S2). *Gabbr1* encodes an essential subunit required for GABA_B_R activation. As shown in Figures 3A,B, dual treatment with GLP-1RA and baclofen significantly shortened the action potential duration at APD90 (MAPs, 150 ms) in wild-type mice. This effect was abolished in *Gabbr1* ^cKO^ mice and in those receiving monotherapy with either semaglutide or baclofen (Figures 3G,H). We next evaluated the ALT in the left ventricle by S1–S1 pacing. In Gabbr1-intact mice (Figures 3C,D), combined semaglutide and baclofen treatment attenuated MI-induced ALT prolongation. In contrast, no changes in ALT were observed in *Gabbr1* ^cKO^ mice under either monotherapy or combination therapy (Figures 3I,J). To further assess arrhythmic susceptibility, we performed burst pacing (Figures 3E,F). In Gabbr1^f/f^ mice, co-administration of GLP-1RA and baclofen reduced both the incidence and duration of post-MI ventricular arrhythmias. However, in *Gabbr1* ^cKO^ mice, dual GLP-1R/GABA_B_R activation failed to confer anti-arrhythmic protection (Figures 3K,L). Collectively, these findings demonstrate that GABA_B_R activation is required for the anti-arrhythmic effects of GLP-1RAs.

### 3.4 GABA_B_R is required for the cardiac function improvement relative to GLP1RA

**FIGURE 4:**
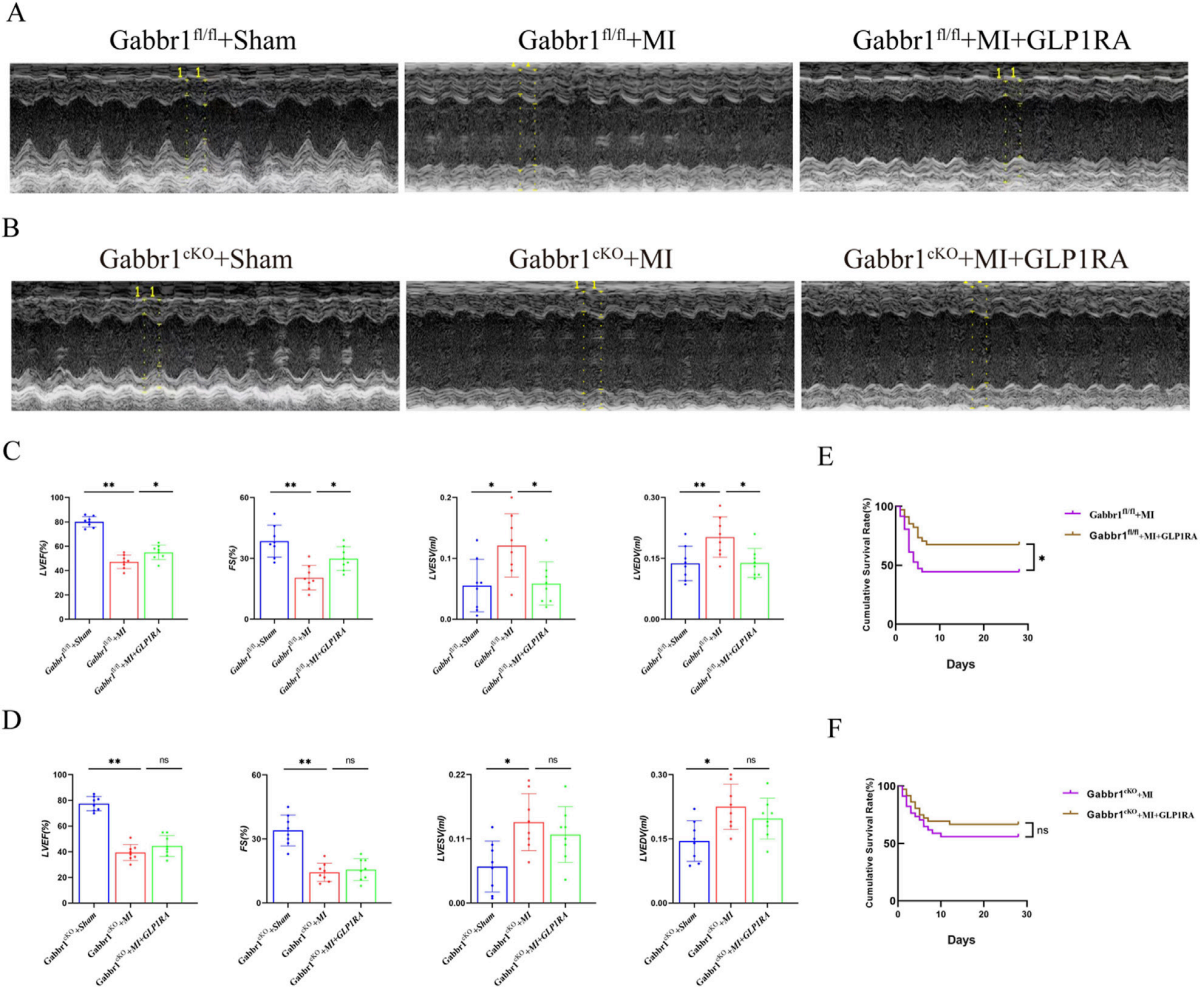
GABABR cardiomyocyte knockout counteracts cardiac dysfunction relative to GLP-1RA. **(A,B)** Representative images of left ventricular M-mode echocardiographic recordings. **(C,D)** Quantitative analysis of cardiac function by LVEF (%), LVFS (%), LVEDV (mL), and LVESV (mL) in all groups (n = 8 biological replicates). **(E,F)** Kaplan–Meier survival curves of the MI group and MI + GLP-1RA group mice 4 weeks after MI (n = 34 per group). Data are presented as mean ± SD. Differences among more than two groups were compared using ANOVA followed by Tukey’s test. SD, standard deviation. *P < 0.05 and **P < 0.01.

To determine whether dual activation of GABABR and GLP-1R further improves cardiac function, we performed echocardiography in *Gabbr1* ^f/f^ and *Gabbr1* ^cKO^ mice after MI. In wild-type mice, GLP-1RA administration improved post-MI cardiac performance, as reflected by increased LVEF and LVFS and reduced LVESV and LVEDV (Figures 4A,C). In contrast, *Gabbr1* ^cKO^ mice failed to show any improvement in cardiac function or survival following GLP-1RA treatment (Figures 4B,D). Moreover, GLP-1RA reduced the 30-day post-MI mortality rate in wild-type mice, whereas this benefit was abolished in *Gabbr1* ^cKO^ mice (Figures 4E,F). Together, these results indicate that GABABR activation is essential for the cardioprotective and survival benefits of GLP-1RA therapy after MI.

### 3.5 GABA_B_R/GLP-1R signaling has no acute effect on fibrosis

**FIGURE 5:**
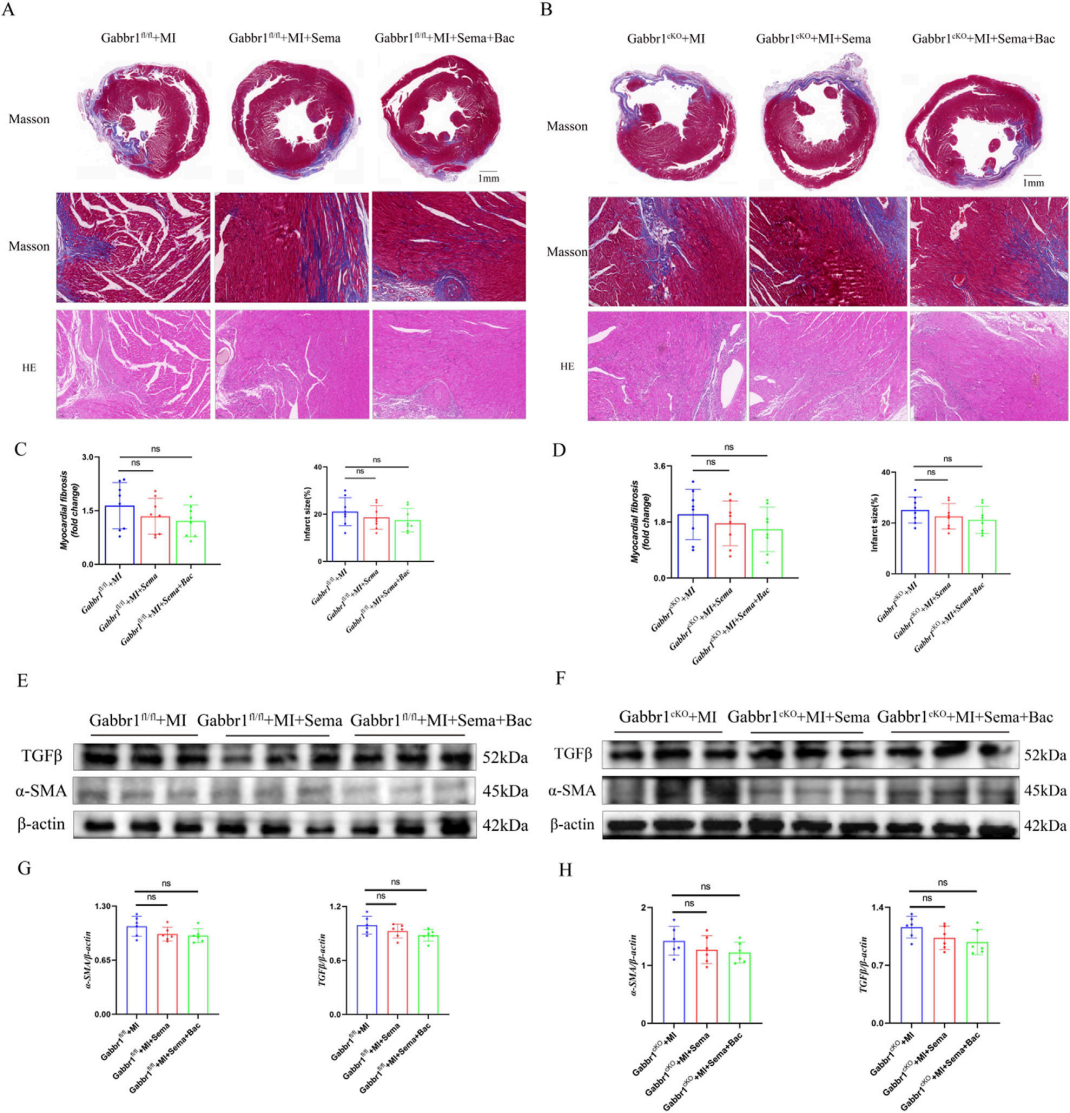
GABABR/GLP-1R signaling has no acute effect on fibrosis. **(A,B)** Representative images of Masson’s trichrome and HE staining. **(C,D)** Quantitative analysis of relative fibrosis area in the groups (n = 8 biological replicates); **(E–H)** Representative Western blotting bands and quantitative analysis of α-SMA and TGFβ in all groups (n = 8 biological replicates). Data are presented as mean ± SD. Differences among more than two groups were compared using ANOVA followed by Tukey’s test. SD, standard deviation; MI, myocardial infarction; Sema, semaglutide; Bac, baclofen *P < 0.05 and **P < 0.01.

To further assess the role of GABA_B_R/GLP-1R dual activation in post-infarction remodeling, we examined cardiac fibrosis using Masson’s trichrome and HE staining. *Gabbr1* deficiency did not alter the cardiac morphology or structure after MI, regardless of treatment with semaglutide alone or in combination with baclofen (Figures 5A,B). Quantitative analysis showed no significant differences in collagen deposition or infarct size with GLP-1RA treatment, either alone or with GABA_B_R co-activation (Figures 5C,D), indicating that neither GLP-1R nor GABA_B_R activation confers acute antifibrotic benefit. Consistently, Western blotting of left ventricular tissue revealed no changes in α-smooth muscle actin (α-SMA, a marker of myofibroblast activation) or transforming growth factor-β (TGF-β, a pro-fibrotic marker) in either wild-type (Figures 5E,G) or *Gabbr1* ^cKO^ mice (Figures 5F,H). Collectively, these findings suggest that acute GABA_B_R/GLP-1R activation does not significantly modulate cardiac fibrosis progression after MI.

### 3.6 GABA_B_R-induced inward-rectifying current is inhibited by GLP-1RA

**FIGURE 6:**
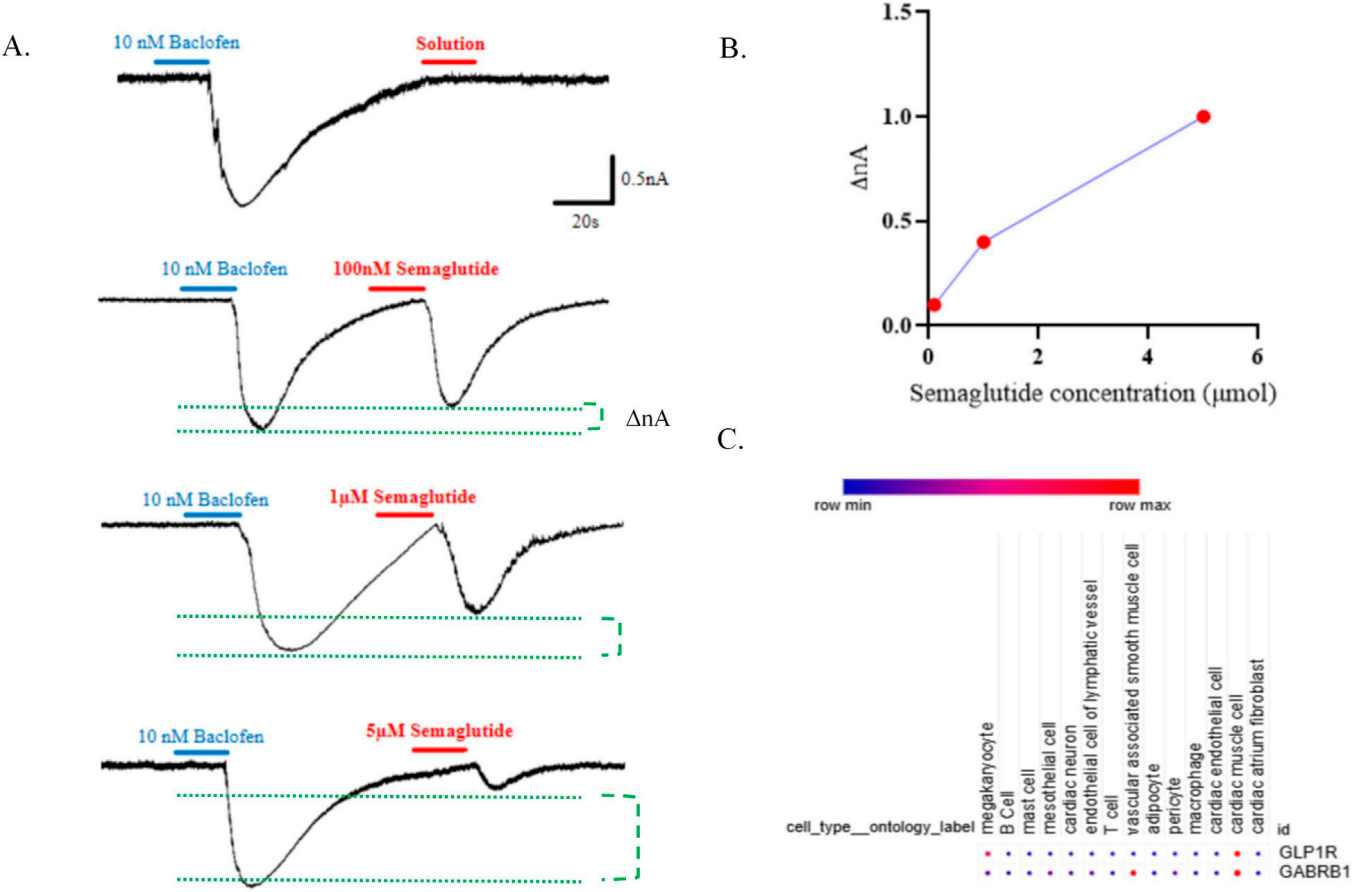
The extracellular GLP-1R domain is sufficient for modulation of GABAB receptor-gated inward-rectifying current. **(A)** Currents evoked by baclofen (10 nM) and semaglutide at 100 nM, 1 μM, and 5 μM in iPSC-derived cardiomyocytes. **(B)** Quantification of the dose–current relationship. **(C)** Cell specificity of GLP-1R and GABABR expressions.

GABA_B_R activation enhances potassium conductance through inwardly rectifying GIRK channels in a G protein-dependent manner, and baclofen is sufficient to induce this current. To assess whether GLP-1RAs modulate GABA_B_R-mediated inward currents, we performed whole-cell patch-clamp recordings in hiPSC-derived cardiomyocytes. Baclofen evoked inward currents, which were suppressed by GLP-1R activation in a concentration-dependent manner (Figures 6A,B). In addition, re-analysis of single-cell RNA sequencing data from cardiac tissue of 46 arrhythmia patients (Matthew C. Hill et al.) revealed co-expression of GABABR and GLP-1R in cardiomyocytes (Figure 6C) (Marso et al., 2016). Together, these findings suggest that GLP-1RAs attenuate GABABR-gated depolarizing currents in cardiomyocytes.

## 4 Discussion

The positive chronotropic effect of GLP-1RAs are well documented, yet their mechanism remains elusive (Yang et al., 2024; Lu et al., 2023). To determine whether GLP-1RA acts via neural-dependent or neural-independent pathways is critical for optimizing GLP-1RA usage in cardiac ischemic diseases. Although neuronal GLP-1R is concentrated in autonomous neuron axons and synaptic regions, cardiomyocyte autonomous GLP-1R appears to work independently. Our study demonstrated that sympathetic denervation did not abolish the GLP-1RA-mediated modulation of the HR in MI mice. This contrasts with previous models attributing GLP-1RA-induced tachycardia to the autonomous nervous system as our findings suggest an cardiomyocyte-expressed GABA_B_R-involved mechanism.

GABA_B_R signals via G-proteins and is localized at pre- and post-synaptic sites. Presynaptic GABA_B_Rs couple to Ca^2+^ channels to regulate neurotransmitter release, whereas postsynaptic GABA_B_Rs activate inwardly rectifying K^+^ (Kir3) channels, mediating slow inhibitory currents (Lorente et al., 2000; Fortin et al., 2020). Our data indicate that postsynaptic GABA_B_Rs in cardiomyocytes interact with GLP-1R to modulate spontaneous membrane potential, implicating a potential antiarrhythmic role following cardiac ischemia. The interaction between GLP-1R and GABA_B_R has also been characterized in the central nervous system. For instance, GLP-1R knockout in the nucleus tractus solitarius attenuates GABAergic signaling, reducing food intake and body weight (Jagomäe et al., 2025). Additionally, local liraglutide administration suppresses postsynaptic GABA receptor activity while enhancing presynaptic GABAergic neuron firing (Maitre et al., 1983). These findings suggest that GLP-1R signaling modulates GABA receptor function. Notably, both γ-hydroxybutyrate, a GABA analog, and the GABA_B_R agonist baclofen induce dose-dependent hyperpolarization via Kir channel-mediated K^+^ efflux, supporting functional GABA_B_R expression in cardiomyocytes (Dauvilliers et al., 2022; Ast et al., 2023). To further validate this mechanism, we generated cardiomyocyte-specific GABA_B_R-knockout models. Intriguingly, GABA_B_R deletion led to GLP-1R-mediated HR elevation, whereas GABA_B_R activation with baclofen reduced the HR. These findings suggest a neural-independent mechanism by which GLP-1R-GABA_B_R interaction regulates cardiac electrophysiology.

So far, the therapeutic potential of GLP-1RAs in ischemic myocardium has been debated. Although some clinical trials reported a reduced myocardial injury biomarkers and modest improvements in left ventricular ejection fraction, others showed no significant outcome benefits. Furthermore, GLP-1RA-induced tachycardia may increase myocardial oxygen demand, potentially offsetting metabolic benefits in acute ischemia. Our data propose that selectively blocking GLP-1R-mediated chronotropic effects via agonizing GABA_B_R might expand GLP-1RA utility into acute coronary syndromes. In addition, the effect of GLP-1RA on the HR is consistent at both the early phase (autonomic overdrive) and late phase (recovered innervation) of MI, further supporting the phenomenon of autonomous nerve independence. GLP-1RA is believed to mediate intracellular signaling through Gαs, thus activating subsequent adenylate cyclase and increasing the cAMP level (Durak and Turan, 2023). As a result, protein kinase A is activated. Acute cAMP/PKA enhancement can mediate the positive chronotropic effect and attenuation of calcium entrance and RyR2 phosphorylation to cause arrhythmogenesis (Younce et al., 2013; Yaniv et al., 2015). Meanwhile, the cAMP/PKA cascade is positioned in the negative regulation of adenylate cyclase by GABA_B_R (Lubberding et al., 2024). Otherwise, after GABA_B_R activates inward-rectifying channels, the outward K^+^ current increases, leading to hyperpolarization of sinoatrial node cells and counteracting the depolarization caused by GLP-1R through HCN channels (I_f_ current) to reduce the pacing frequency. Moreover, inhibiting the action potential increasing rate via activation of L-type calcium channels (I_Ca-L_) could also contribute to this effect (Lüscher et al., 1997).

GLP-1R agonists, whether administered centrally or peripherally, can increase the HR in rodents (Wei and Mojsov, 1995; Wei and Mojsov, 1996; Bullock et al., 1996), induce the expression of c-Fos in adrenal medullary catecholamine neurons, and activate tyrosine hydroxylase in the brainstem. We believe these data suggest that some of the rapid cardiovascular effects of GLP-1 may be due to the increased outflow of catecholamines in the brain and elevated sympathetic nerve tension. Under physiological conditions, GLP-1RA’s fast HR effect can be dominated by the autonomous tone. However, under denervating conditions, cardiomyocyte-autonomous GLP-1R can also be a compensative mechanism under the assistance of some co-receptors, such as GABA_B_R. However, a previous study by Lubberding et al. showed that denervation could not abolish the regulation of GLP-1RA on the HR (Lubberding et al., 2024), which is consistent with our findings. There are some limitations to our work. The distribution pattern of GLP-1R differs between mice and humans, which may result in a difference in the mechanism, as GLP-1R in humans is more expressed on cardiomyocytes, whereas Glp-1r in mice is expressed more on endothelial-like cells from single-cell data (McLean et al., 2022). We have yet to consider the expression pattern of GLP-1R between human and mice, which may limit the clinical relevance. Our study focuses on the overall effect of GLP-1RA without GLP-1 analogs for a positive control and provides no evidence to illustrate that the vascular expressed Glp-1r is not important for the chronotropic effect and anti-arrhythmia potential of Gababr/Glp-1r dual agonization. Finally, baclofen, administered 1 day before semaglutide, likely has central effects (and residual sedation) with a half-life of approximately 3 h. The HR was measured under isoflurane, which can also depress heart beating and modulate the autonomic tone. In the future, whether GLP1 directly synergizes with GABA_B_R activation or GLP-1R activation sensitizes GABABR signaling remains to be tested. The relationship between postsynaptic GLP-1R activation and presynaptic GABA release requires further exploration.

Overall, our findings identified a novel cardioprotective axis, where GLP-1R-GABA_B_R cross-talk fine-tunes cardiac electrophysiology, and under denervating conditions, cardiomyocyte GLP-1R can also be a compensative mechanism under the assistance of some chaperones such as GABA_B_R.

## Data Availability

The original contributions presented in the study are included in the article/Supplementary Material, further inquiries can be directed to the corresponding authors.
