# Supplementary figures and images for: Agonizing GABABR suppresses GLP-1RA’s chronotropic effect and reduces post-myocardial infarction arrhythmogenesis
Source: Front Pharmacol. 2025 Oct 31;16:1616181. doi: 10.3389/fphar.2025.1616181 (PMC12616031; doi:10.3389/fphar.2025.1616181)

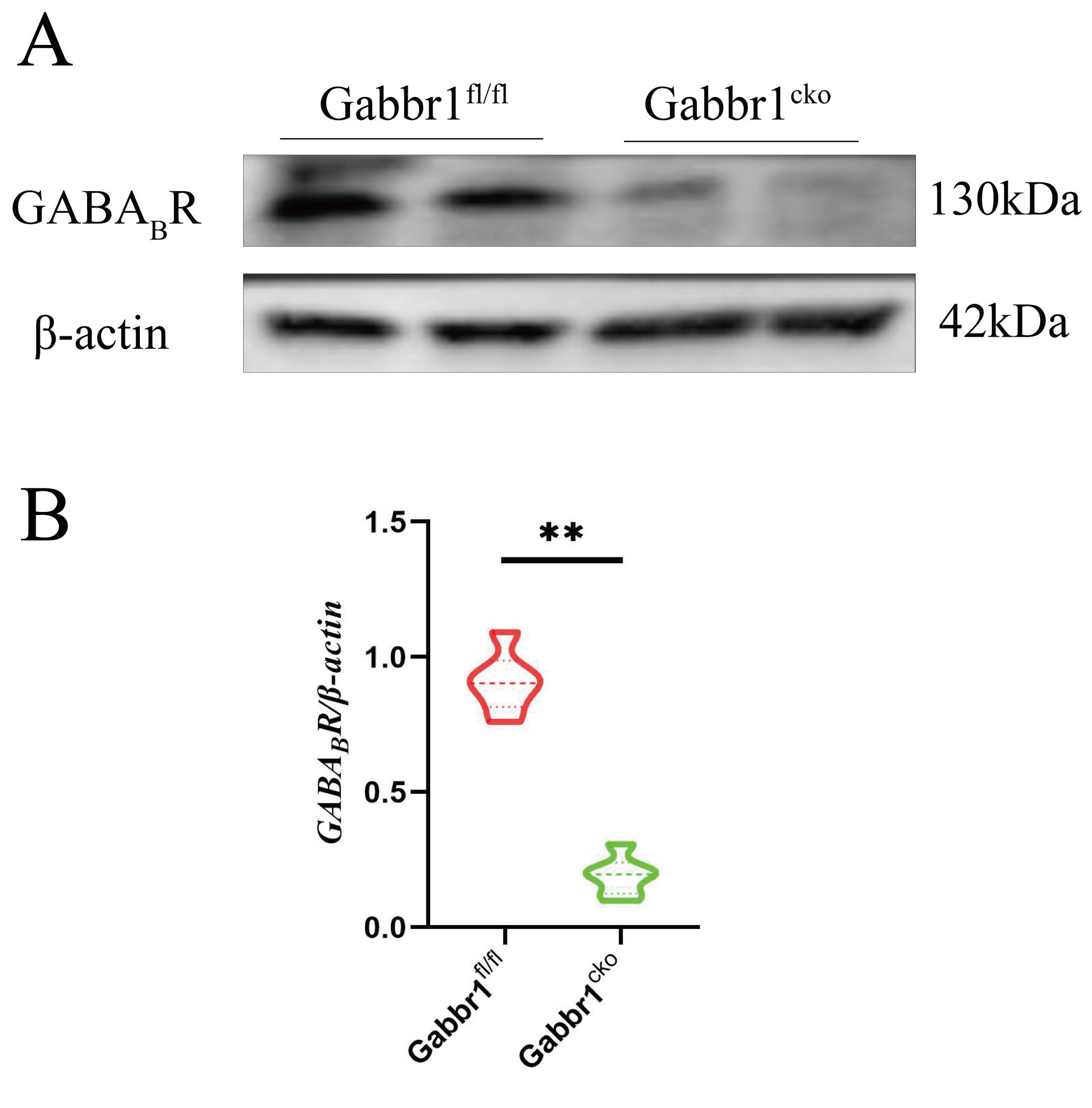

Supplement: Supplementary file 1 [file Image2.tif]

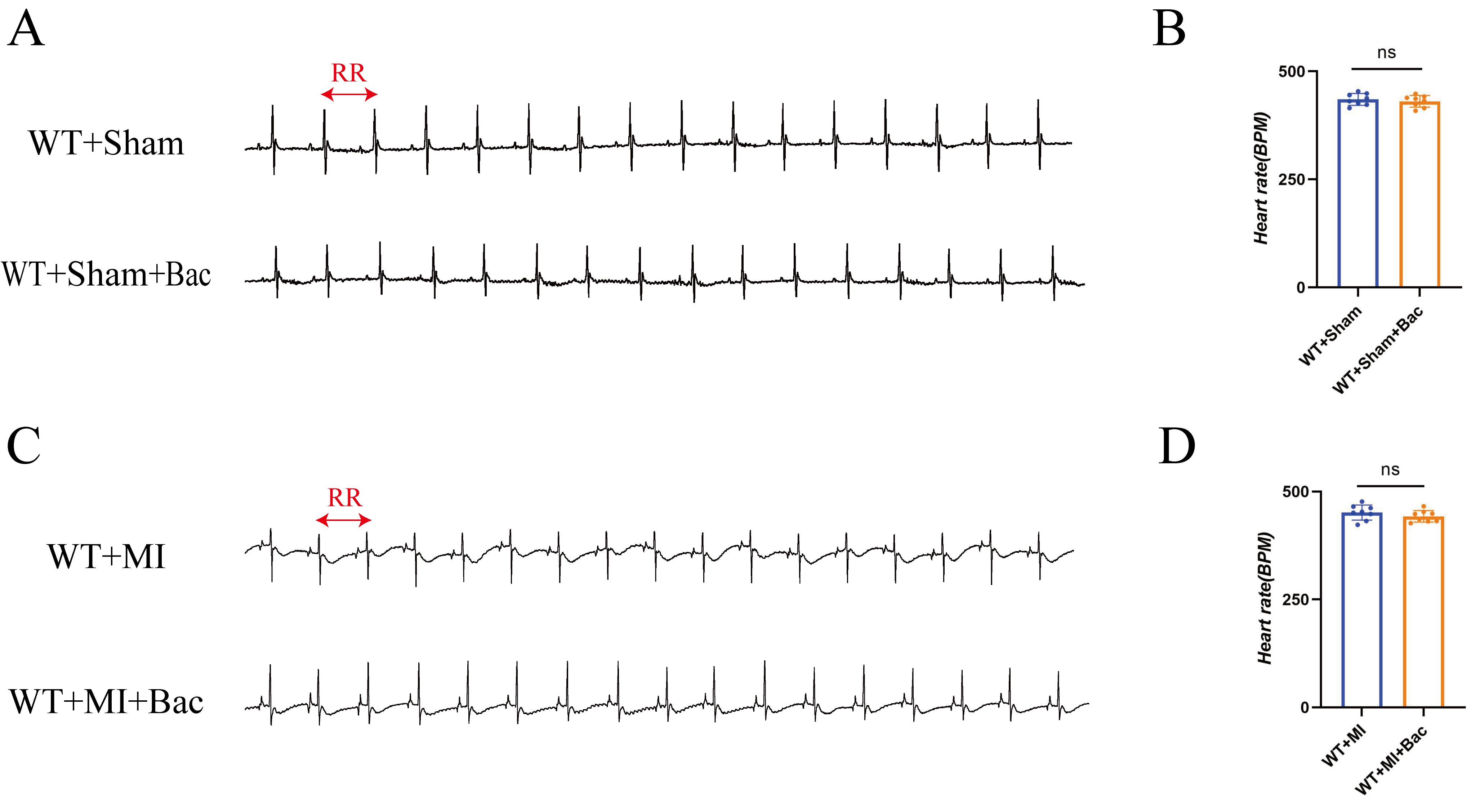

Supplement: Supplementary file 2 [file Image1.tif]
